# Endogenous Endophthalmitis—The Clinical Significance of the Primary Source of Infection

**DOI:** 10.3390/jcm11051183

**Published:** 2022-02-23

**Authors:** Małgorzata Gajdzis, Kornelia Figuła, Joanna Kamińska, Radosław Kaczmarek

**Affiliations:** Department of Ophthalmology, Wroclaw Medical University, 50-367 Wroclaw, Poland; kornelia.figula@gmail.com (K.F.); kaminska.joanna.ewa@gmail.com (J.K.); radoslaw.kaczmarek@umed.wroc.pl (R.K.)

**Keywords:** endophthalmitis, endogenous endophthalmitis, ocular infection, bacterial endophthalmitis, fungal endophthalmitis

## Abstract

Endophthalmitis is a severe form of ocular inflammation. The source of pathogens in endogenous endophthalmitis is located inside the body, and infection spreads hematogenously. Although rare, endogenous endophthalmitis is a very serious condition, as this type of inflammation is very devastating for ocular tissues. Prognosis is very poor, and the patients are often in a serious general condition, so they require special care and an individual approach in the treatment process. Thanks to the knowledge of the risks associated with infections of individual tissues and organs as well as potential pathogens and the clinical picture, it is possible to make a correct diagnosis faster and implement the correct treatment. In the case of endogenous endophthalmitis, reaction time is absolutely crucial for prognosis. In this review, we focus primarily on the importance of the primary source of infection for the course of the disease and prognosis.

## 1. Background

Endophthalmitis is a severe form of ocular inflammation. It occurs when infecting organisms enter the posterior segment of the eye. Depending on the pathway by which microbes enter the eye, we distinguish between two basic forms of inflammation. The source of pathogens in the exogenous endophthalmitis is the ocular surface or the environment, and their entry into the eye occurs by inoculation (postoperative, after intravitreal injections, keratitis-related, bleb-related, device-related, or post-traumatic). In endogenous endophthalmitis, the source of infection is inside the body, and infection spreads hematogenously. Endogenous endophthalmitis (EE) is much less common, accounting for about 5–15% of cases [1]. However, in different reports proportions ranged widely due to geographic, genetic or alimentary factors [2].

Endophthalmitis is very devastating for ocular tissues. The retina is particularly susceptible to the negative effects of inflammation because it has little ability to regenerate. Damage occurs rapidly, therefore, in order to improve the prognosis, quick and accurate diagnosis and effective treatment are extremely important. An incorrect initial diagnosis (usually as uveitis) may cause a delay in therapy. According to the available literature, errors in diagnosis may concern up to 25–33% of cases [3,4]. Damage of ocular tissues is caused by pathogens as well as by the immune response, and is exacerbated by the ischemia caused by septic emboli [5]. For this reason, treatment must include both removing pathogens from the eye and inhibiting intense inflammation.

In order to limit the destructive effects of inflammation, the eye has a specific relationship with the immune system. This phenomenon is called immune privilege. One of the better understood immune privilege mechanisms is the blood–retina barrier. It limits the unrestricted entry of blood cells and large molecules into the eye. Another contributing factors are absence of lymphatic drainage pathways and inhibitory ocular microenvironment, consist of soluble and cell-bound immunomodulatory factors [6]. Resident immune cells also play a vital role in the immune response. They participate in destroying pathogens without damaging ocular tissue and also inhibit immune cell infiltration [7]. However, due to dysfunction of the blood–retina barrier caused by the pathogens, ocular immunosuppression cannot inhibit non-resident immune cells from infiltration. Although the first symptoms of infection occur thanks to non-resident cells, which allows for its diagnosis and treatment, it is the action of these cells that is responsible for the destruction of extremely delicate eye tissues [7]. Animal studies show that untreated EE leads to profound changes in the structure of the retina, and even when bacteria are removed, inflammation continues, damaging the tissues [8]. In connection with attempts to repair the breakdown of the blood–retinal barrier, some researchers suggest the use of corticosteroids in the course of EE. However, such a procedure is controversial [9]. In extreme cases, in the event of therapy failure, enucleation may be necessary. The histopathological examination of the removed eyes also shows deep inflammatory changes, infiltration of tissues by inflammatory cells, and their disorganization [10].

Age, sex, and race are irrelevant to the risk of developing EE. The disease can develop bilaterally, but most cases (over 70%) affect one eye [11]. Interestingly, inflammation occurs more often in the right eye. This phenomenon is attributed to the peculiarities of the circulatory system, with more direct blood flow from the heart to the right carotid artery [2]. Immunodeficient conditions such as diabetes, cancer, and chemotherapy increase the risk of developing EE. They are associated with reduced host defenses and a higher incidence of infections that can become a source of EE [5]. Diabetes mellitus is the most common disease predisposing to the development of EE [2,12,13,14]. The most probable cause is a dysfunction of the blood vessels leading to an increasing permeability of the blood–retinal barrier. Moreover, elevated blood glucose levels facilitate the multiplication of microorganisms [15]. Disease control is particularly important for the prognosis. The incidence of EE has been shown to be 0.47-fold lower in patients with properly treated diabetes [16].

Only half of EE patients have symptoms of an underlying infection at diagnosis. For this reason, if EE is found in an apparently healthy patient, a high degree of suspicion should be exercised, and the source of infection should be carefully searched for [1]. As systemic symptoms may be non-specific (fever, malaise, abdominal discomfort, pain), infection foci can be easily missed, allowing the progression of a dangerous, often life-threatening disease. On the other hand, in severely ill patients, ocular signs and symptoms are often disregarded [2]. There have been many reports on the incidence of EE in patients with bacteremia and fungemia. The risk of a hospitalized patient developing EE from bacteriemia or fungemia is low, 0.05–0.4% [17]. The question of the validity of routine ophthalmic examination remains open. It is believed that in patients with fungemia they should be performed, as in patients with Klebsiella bacteremia and in other cases in the presence of risk factors for the development of EE [18].

## 2. Primary Source of Infection

In most cases, the primary source of the infection is easy to diagnose as inflammation causes symptoms such as pain, fever, or malaise. However, the symptoms may be not very severe. They can also be masked by the medications taken by the patient-mainly over-the-counter painkillers and anti-inflammatory drugs. Identifying the primary source of infection is important as most of these conditions require intensive and rapid treatment as life-threatening conditions. Moreover, having knowledge of the primary source, it is possible to predict the type of causative pathogen with high probability and promptly implement empirical treatment.

### 2.1. Pyogenic Liver Abscess

Liver abscess is a rare disease, but it is associated with high mortality, up to 15% [19]. Incidence varies depending on the region of the world. In the US and Western countries, it is currently estimated to be 4.1 per 100,000, in Canada, the United Kingdom and Denmark it is 1.1–2.3 per 100,000. In Asia, the incidence is much more frequent, from 5.6 per 100,000 in mainland China, to 17.6 per 100,000 in Taiwan. Patients with comorbid conditions, especially with diabetes, malnutrition, and immunosuppression, are at greater risk [20]. Pyogenic liver abscess is currently most often a complication of inflammation of the bile ducts; less often the cause is seeding of the portal vein from an intra-abdominal infection (appendicitis, diverticulitis, inflammatory bowel disease), direct extension (from cholecystitis, perinephric abscess, and subphrenic abscess), hematogenous abscess spread, trauma, or postoperative complications. Interestingly, about 30% of cases are cryptogenic [20]. The most common symptoms of pyogenic liver abscess are fever and pain in the right upper abdomen [21]. The organisms most frequently isolated during infection are Streptococcus species, Klebsiella pneumoniae and Escherichia coli. Occasionally, cases of other etiology are also reported [22,23].

Liver abscess due to Klebsiella was described in Taiwan in the 1990s. Currently, it is the most frequently isolated pathogen in liver abscess in Asian countries, and its incidence is also significantly increasing in the countries of Europe and North America [19]. This trend is especially relevant from the point of view of ophthalmologists because liver abscess due to Klebsiella is associated with the metastatic spread (in 12% of cases, compared to <1% in non-Klebsiella etiologies) [1,19]. The most common manifestations are meningitis and endophthalmitis, however bacteremia, pneumonia, splenic abscess, and necrotizing fasciitis may also occur [24]. The actual incidence of EE in Klebsiella liver abscess ranges from 0.9 to 7.9% [25]. Liver abscess is also the most common extraocular focus of infection in EE in Asia [2]. In a large systematic review of 342 cases of bacterial EE liver abscess was the most common source of infection worldwide (in 19%) [4]. In a retrospective review of EE presenting data from 2009 to 2016 at a referral center in south India, liver abscess was the common concomitant infection (in 14.7%) [12].

From a diagnostic point of view, it is extremely important that over 16% of Klebsiella patients with EE have negative blood culture results. Furthermore, the risk of developing EE due to a liver abscess is not related to the presence of diabetes mellitus. What is more surprising, the prevalence of diabetes mellitus remains significantly higher in patients with Klebsiella liver abscess as compared to the patients with non-Klebsiella etiology [25].

**Figure 1:**
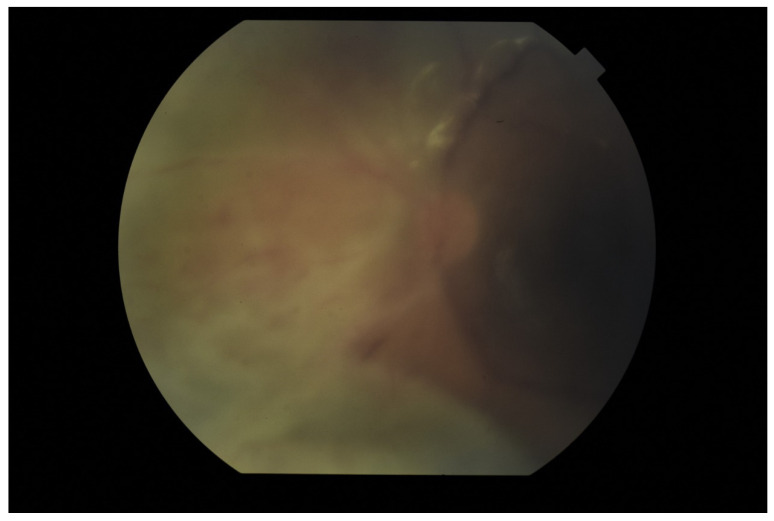
Fundus pictures of subretinal abscess, taken on the second day after vitrectomy. Partially drained abscess is visible, located nasally from the optic disc. Poor image quality is caused by the presence of inflammatory cells in the anterior chamber.

**Figure 2:**
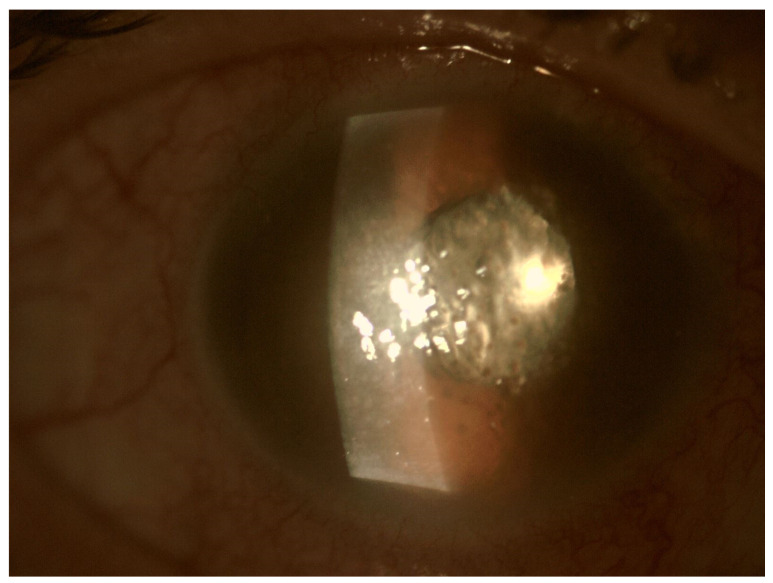
Pre-operative photo of the anterior segment of the eye in the patient presented in Figure 1. Attention is drawn to the widening of the conjunctival blood vessels, damage to the corneal epithelium and cell deposits on the endothelium. Due to the poor transparency of optical centers, the view into the fundus was significantly impaired.

The tendency to abscess formation in the course of Klebsiella infection is also reflected in eye. One of the rare but characteristic feature of EE in the course of Klebsiella infection is the formation of subretinal abscesses [26]. They take the form of retinal elevation and paleness (Figure 1 and Figure 2). With properly selected antibiotic therapy, even large abscesses may be absorbed. If the patient is undergoing vitrectomy, intraoperative drainage may be considered [27].

The prognosis of EE in the course of liver abscess is poor. Many reported cases end in enucleation or a lack of sense of light [4,23]. A good visual outcome is possible only in the case of a very rapid implementation of intensive treatment. The analysis of cases published in the years 1986–2012 indicates that better treatment outcomes were achieved in patients undergoing vitrectomy compared to those treated only with intravitreal injections of antibiotics. Visual prognosis seems to improve in recent years, now 41% of eyes have achieved visual acuity of 20/200 or better. However, still 19% of patients required enucleation or evisceration [4].

### 2.2. Endocarditis

Infective endocarditis (IE) is a life-threatening infection of the endocardium. The inflammatory process can affect the heart valves, resulting in their destruction and formation of abscesses. This condition may lead to valvular dehiscence and acute heart failure [28]. Among hospitalized patients with hematogenous infections in the United States, infective endocarditis is one of the most common risks for the development of EE. In Europe, endocarditis is the third most common infectious source in patients with EE [29]. In case of EE caused by group B Streptococcus endocarditis is the most common non-ocular infection focus [11]. In studies from centers in India, endocarditis was associated with 5.8% of EE cases [12].

The pathogen responsible for the most cases of endocarditis is Staphylococcus aureus-up to 40%. It presents a great therapeutic challenge due to the frequent resistance to antibiotics and a predilection for acute complications such as stroke. Other common endocarditis pathogens are viridians group streptococci and enterococci in case of native valve infection, and Coagulase-negative staphylococci in case of prosthetic valves and cardiac devices infection. Gram-negative bacteria and fungi are responsible for a smaller but growing number of cases [30].

EE is rarely the first symptom of endocarditis, as it usually indicates a developed infection that is difficult to overlook or ignore, but such cases have been reported [28,31]. As endocarditis is a common cause of EE, a transesophageal echocardiogram should be performed in any patient with an unknown source of infection and suspected heart failure. Transthoracic echocardiogram is not recommended to rule out endocarditis [28]. Patients with EE and endocarditis has a bad prognosis for visual acuity, almost 60% finally lost all vision. Mortality is also high: 8–24% depending on the study [11].

### 2.3. Meningitis

Bacterial meningitis is a life-threatening infection due to central nervous system complications. It is mainly caused by Streptococcus pneumoniae and Neisseria meningitidis, the latter in the younger population [32,33]. EE can be a first symptom of bacterial meningitis and bilateral cases are reported quite frequently [32,34]. EE can also develop due to Candida meningitis, cases have been reported in immunocompromised people, including children [35]. In general, among bacterial EEs, meningitis is a source of infection in about 2–6% of cases [4,14].

The prognosis for patients with EE in the course of meningitis is poor. Patients with concomitant meningitis and EE are usually in a severe general condition. They are often unconscious, so the developing eye inflammation can be easily overlooked, delaying the initiation of proper therapy. Moreover, the pathogens causing EE has specific characteristics that make them more dangerous to the eye tissue. Streptococcus pneumoniae secretes exotoxins and enzymes which destroy the cellular membranes of the iris and ciliary epithelial cells. This in turn leads to cellular destruction, hypotony, and anterior segment necrosis [33]. For this reason, the damage to the eye is exceptionally large, even with relatively good response to the antibiotics used. In the published literature, most EE cases of Streptococcus pneumoniae etiology eventually lose light perception [33]. Neisseria meningitidis, on the other hand, is difficult to isolate from cultures of eye material, which significantly delays diagnosis. Moreover, as a rare disease, often with an atypical course, it causes additional difficulties in diagnosis and treatment [36,37,38].

### 2.4. Urinary Tract Infection, Urological and Gastrointestinal Interventions

The genitourinary system is the common way by which pathogens gain access to the bloodstream. Severe infections usually occur in people with complicated urinary tract infections and after urological procedures in which adequate perioperative prophylaxis is not used. A wide spectrum of pathogens is isolated in the cultures. The most common are *E. coli*, Candida, Enterococci and Klebsiella. Each of these organisms can cause EE by dissemination [39,40].

The most common metastatic sites from urinary tract infection are the skeleton and the endocardium. EE can also develop, especially in immunocompromised patients [41]. Urological interventions along with gastrointestinal are the most common predisposing factor to the occurrence of fungal EE, especially those of Candida etiology [42]. In large systematic review of 342 cases of bacterial EE urinary tract was the source of infection in 6% of patients [4]. In a retrospective review of EE presented in 2009–2016 at a reference center in southern India, urinary tract infection was the most common coexisting infection, occurring in 32.5% of cases [12]. Urinary tract infection was also the most common source of bacteremia in patients with EE at a tertiary referral hospital in Malaysia (17.5% of cases) [43]. In Denmark, in 2000–2016, urinary tract infection was the source of EE in 10% of cases [14].

Isolated EE rarely develops in urinary tract infections. Patients most often suffer from sepsis and the infection affects many places in the body, such as aortitis, splenic abscess, arthritis, or osteomyelitis. It is also not uncommon to cause inflammation of the soft tissues of the orbit [44]. Urine culture is particularly helpful in the presence of dysuria, which may suggest that the primary source of infection may be the urinary tract. Unlike blood cultures it is positive in most cases [45].

A greater risk of EE development is associated with urological surgery, also minimally invasive [46]. One of the most important reasons is the frequent coexistence of diseases predisposing to the development of EE, mainly diabetes. Another is the presence of a latent urinary tract infection at the time of surgery (mostly crushing stones). Mechanical injuries, the transfer of microorganisms through the catheter to the upper urinary tract and increased pressure in the renal pelvis during the procedures may contribute to the spread of infection. After some procedures, a stent is left in the urinary tract for 2 to 4 weeks, to drain urine and prevent ureterostenosis. It can also be a source of infection due to chronic irritation of the mucous membranes [15].

Additionally, gastrointestinal procedures are associated with an increased risk of spreading microorganisms through the bloodstream, and thus the development of EE [47]. In a retrospective study in one of the Danish centers carried out in the years 2000–2016, it was shown that complications after intestinal surgery were the source of EE in 6% of cases [14]. Both bacterial and fungal infections have been reported [48,49]. Complications in the form of EE development occur both after extensive surgeries, such as resection of the intestine or stomach, and definitely less strenuous endoscopic procedures [50,51].

### 2.5. Soft Tissue Infections

Soft tissue infection, mainly known as skin abscess or cellulitis, is among the more common causes of EE. In various reports, they account for 6 to more than 18% of the cases of the source of infection [14,43]. Cellulitis and skin abscesses are caused by bacteria, and the most common etiological factors are Group A beta-hemolytic Streptococcus and Staphylococcus aureus [52]. There are also reports in the literature about EE caused by group B streptococcus, where soft tissue inflammation is the most common source of infection, next to the aforementioned endocarditis [53]. As in other sources of infection, the factors predisposing the development of EE in the course of infection of soft tissues are diabetes, intravenous drug use, and immunodeficiency [54].

Various skin inflammations are among the most common complaints reported by patients to primary care physicians [55]. Ophthalmic symptoms may appear shortly after the skin lesions have healed and are sometimes not associated with a history of inflammation [56]. In the case of acne lesions or atopic dermatitis there is no risk of developing EE. However, in the case of treating skin abscess or cellulitis, primary care physicians should alert patients to the risk of this complication. Rapid diagnosis and implementation of effective treatment based on the putative etiology may begin to improve treatment outcomes.

### 2.6. Osteomyelitis

Osteomyelitis is an inflammatory process caused by an infecting microorganism. During inflammation, bone destruction occurs, and the inflammatory process may also affect the bone marrow, the periosteum, and the surrounding soft tissues. The most common etiological factor is Staphylococcus aureus [57]. Group A and B streptococcus, Streptococcus pneumoniae, Kingella, Pseudomonas, Serratia, and Escherichia coli are less common. Fungal infections are very rare and occur only in immunocompromised people [58]. Almost any bone can be the primary site of infection, although the spine, hip, sternum, and ankle are the most commonly described [3,59,60,61].

A large analysis of 342 cases of bacterial EE showed that osteomyelitis accounted for only 0.58% of cases [4]. However, it should be remembered that this is a study based on published cases, which often do not fully illustrate the real scale of the problem. Moreover, some cases can be classified as soft tissue inflammation [62]. In clinical practice, EE coexisting with osteomyelitis is not a rare phenomenon, especially in diabetic patients, where bone changes are chronic.

The prognosis for EE in the course of osteomyelitis is as poor as for other cases. Up to 60% of patients lose their vision completely, and in up to 80% of cases, the preserved vision is limited to hand movements. [59,63].

### 2.7. Intravenous Drug Use

Injecting drug use is a huge problem all over the world. The consequences are extremely serious, ranging from health, through social, and ending with economic. The exact number of drug users is difficult to estimate. Degenghardt et al., in their global multistage systemic review, estimated that globally about 15.6 million people aged 15–64 years inject drugs. In Canada and the United States alone, 2.56 million people (1.06% of the population) are affected. Over 72% declare that opioids are the most frequently used intravenous drug [64]. Another report by Tirpack et al. identifies the area of New England as being particularly hit by the crisis, where the number of deaths related to heroin use has increased significantly since 2014. Consequently, in the years 2014–2016, only one hospital recorded a more than twofold increase in EE cases compared to the years 2012–2014 [65]. In some published case series, up to 50% of EE patients had a history of injecting drug use [66]. There is also an increase in the frequency of other hematogenous infections, such as viral hepatitis, human immunodeficiency virus, infectious endocarditis, or septic arthritis. [65,67]. An analysis of the NIS databases from 2002 to 2014 showed that more than 600,000 patients were hospitalized for infections associated with injecting drug use, 0.1% of them had EE. Patients with EE were more likely to have comorbidities such as diabetes, heart valve disease, kidney damage, cirrhosis, or a history of cancer [67]. Most reports find a higher incidence of EE in men. The source of this relationship is seen in the differences in drug use between men and women. Women are statistically using smaller amounts of drugs and for shorter periods of time than men [68].

An increased risk of developing infections, including EE, in injecting drug users is associated with the direct inoculation of pathogens into the bloodstream during injection. The greatest threat is fungemia, and the most common organism causing EE is Candida [65]. In recent years, infections caused by Rhodotorula spp. have also been observed with increasing frequency [69]. Candidemia increases the risk of developing EE 15 times [67]. In the 1990s, infections of internal organs, including EE, were much more common, which is also attributed to the way drugs are used. Since the 1980s, brown heroin has been mixed with lemon juice to increase its potency. Lemons were very often contaminated with Candida albicans, so the dose injected into the bloodstream was remarkably high. In some reports, up to 50% of candidemia patients had EE [70]. Currently, the prevalence of EE among injecting drug users is around 1% [71].

The diagnosis of infections in the course of intravenous drug use presents additional difficulties, as patients often deny the use of drugs. Of course, the cultures are a hint, but blood cultures may be negative due to transient bacteremia or fungemia [1]. Taking a detailed medical history as well as careful physical examination can be helpful. Medical databases contain numerous case reports of EE in injecting drug users caused by less common pathogens such as Serratia [72,73]. As patients are often in poor general condition, the prognosis is poor.

### 2.8. Indwelling Central Venous Access Devices (CVAD)

Presence of CVAD place patients at risk for different complications—local site infections, bloodstream infections, and thrombosis [74]. Local complications are relatively easy to control, the matogenous infections pose a greater risk. Manifestations are determined by the affected organs. EE is one of the less common complications. Other rare complications are peritonitis, endocarditis, osteomyelitis, and meningoencephalitis. Large retrospective studies from 2000 to 2016 found that EEs associated with the presence of CVAD account for 4% of cases [14].

Bacteria mainly cause the CVAD-related infections (in 86%). The most common cultures are Staphylococcus aureus, less frequently Pseudomonas aeruginosa, Escherichia coli, Klebsiella pneumoniae, Coagulase-negative Staphylococcus spp, and Acinetobacter baumanii. Among fungi, Candida is the most common causative agent (11% of all infections) [75]. EE in the presence of infected CVAD can be caused by any of the above-mentioned microorganisms, but studies show that the main etiological factor of EE are fungi, especially Candida [76]. As with infections associated with intravenous drug use, Rhodotorula infections are increasingly being observed in immunocompromised patients with CVADs. Rhodotorula is ubiquitous microorganism that can be isolated from skin, lungs, and nails. Catheter related fungemia is the leading form of infection and the cause of death of all infections caused by this yeast [69]. Other rare cases of EE are infections caused by Ochrobactrum anthropi or Serratia marcescens [72,77,78].

For CVAD, as in intravenous drug users, blood cultures may be negative due to transient bacteremia or fungemia [1]. In this case, however, the diagnosis is easier to make as the presence of an infected catheter is easy to identify.

## 3. Diagnostic Challenges

The clinical picture of EE can vary significantly, although the main symptoms (decreased visual acuity and pain) and signs (hypopyon and vitritis) remain similar. Proper diagnosis is necessary for the choice of the most appropriate treatment. With EE, misdiagnosis is surprisingly common [3,4]. Due to the similarity of symptoms, it is most often confused with non-infectious uveitis, orbital cellulitis, or conjunctivitis, and in children also with retinoblastoma [4,79]. In order to avoid mistakes, a thorough medical history and physical examination should be carried out. In the case of EE, identifying the source of the infection can bring tangible benefits as some infections are associated with a specific pathogen, such as Klebsiella in the case of a liver abscess. Frequent, regular follow-up examinations are also necessary, as with EE the local condition can deteriorate very quickly.

**Figure 3:**
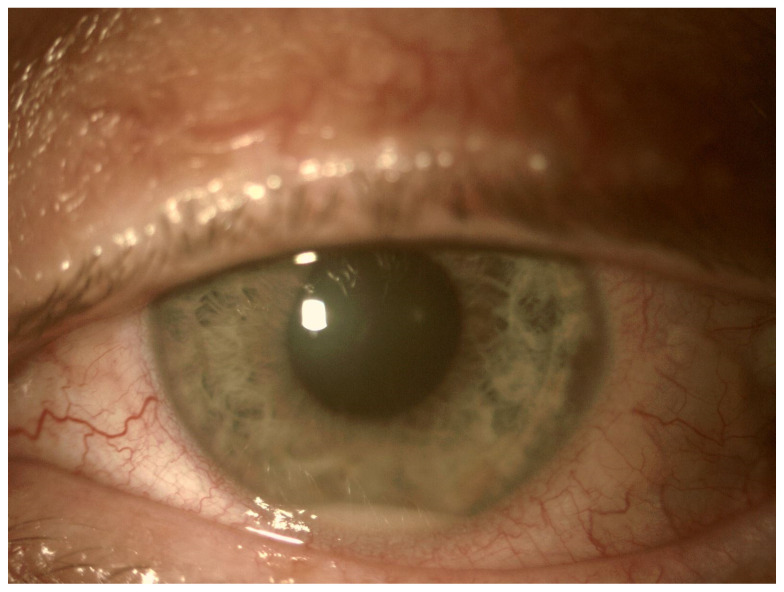
Tyndall in the aqueous humor causes a blurred image of the iris, especially visible below the pupil. In the lower part of the anterior chamber there is a hypopyon.

**Figure 4:**
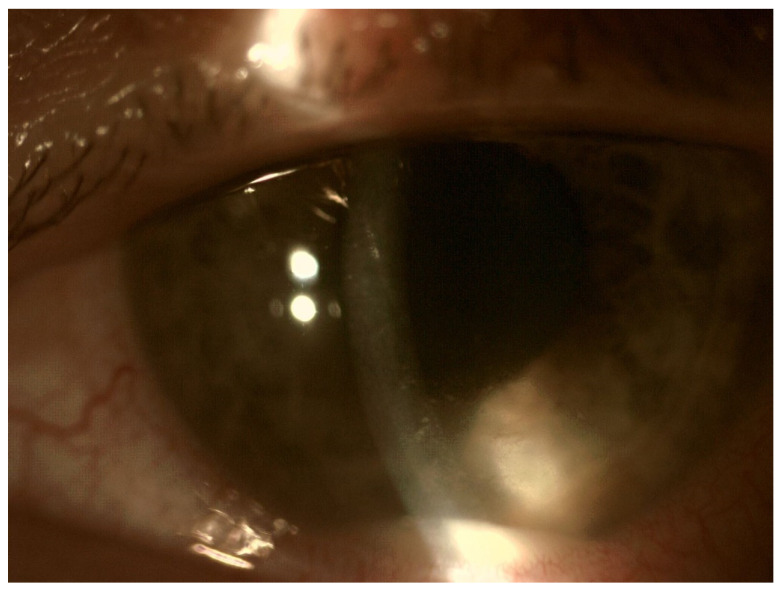
Hypopyon in the anterior chamber. Visible aggregates of inflammatory cells accumulated on the endothelium.

**Figure 5:**
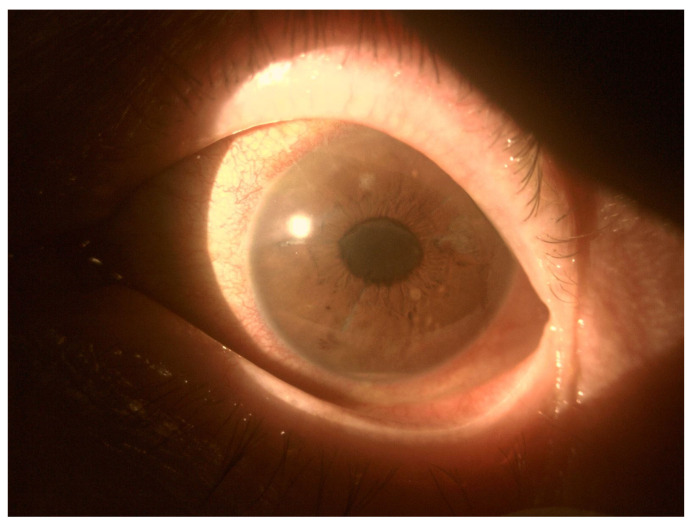
Posterior adhesions causing pupil irregularities. A slight hypopyon and ciliary congestion are also noteworthy.

In EE, the transparency of optical media is often reduced. The cornea opacification and Descemet’s membrane folding are common. Tyndall can be found in the aqueous humor, often there is also hypopyon in the anterior chamber (Figure 3 and Figure 4). Inflammation and bacterial toxins are responsible for cataracts development and leakage of the lens proteins into the anterior chamber. In the case of posterior synechiae, the pupil does not respond to mydriatics (Figure 5).

**Figure 6:**
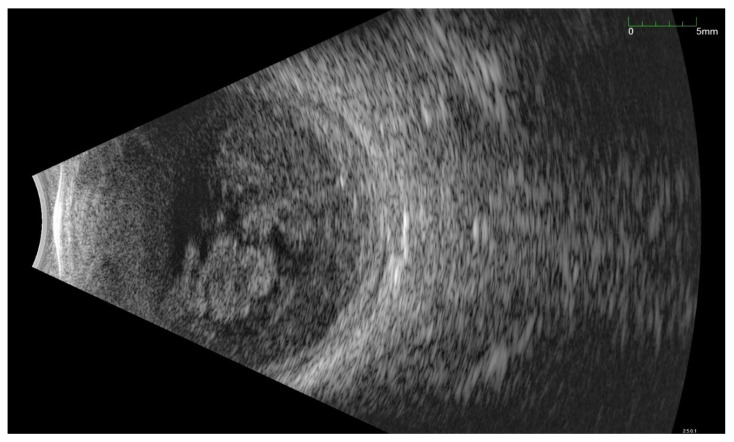
Ultrasound B scan with a hyperechoic exudate filling almost the entire vitreous chamber. Advanced inflammation in a patient with bacterial EE.

**Figure 7:**
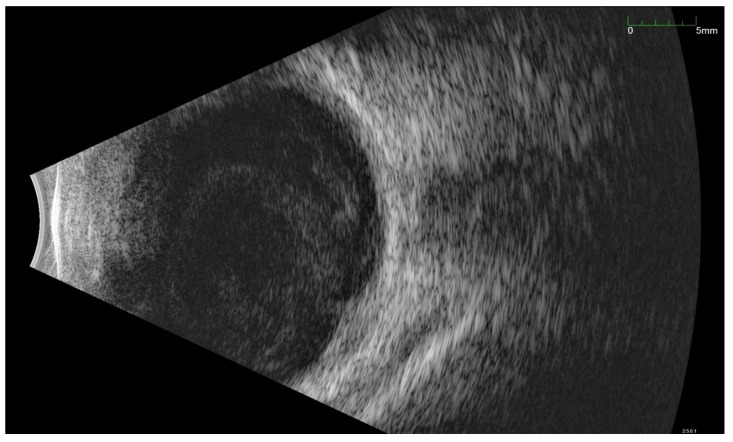
Ultrasonography B scan with bacterial EE. As in Figure 6, hyperechoic densities fill the entire vitreous chamber. However, the lower intensity of the changes is noticeable.

**Figure 8:**
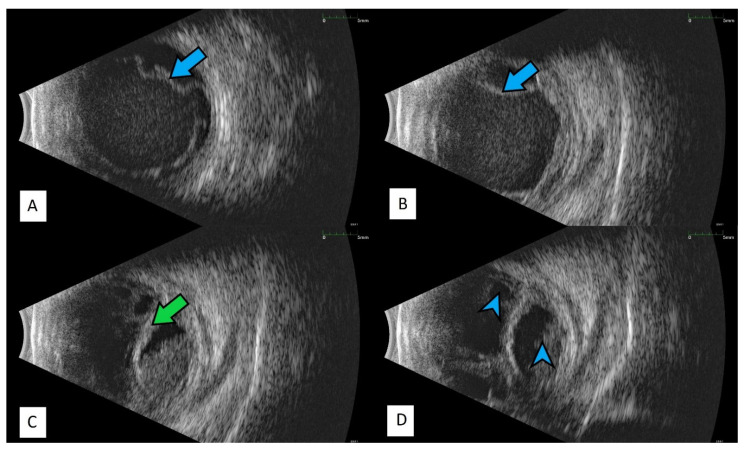
Ultrasound B scans showing the evolution of changes during the development of inflammation. (**A**,**B**)—vitritis, posterior vitreous detachment (blue arrow), and retinal thickening. (**C**,**D**)—retinal detachment (green arrow) and numerous hyperechoic densities in the vitreous chamber (blue arrowhead).

**Figure 9:**
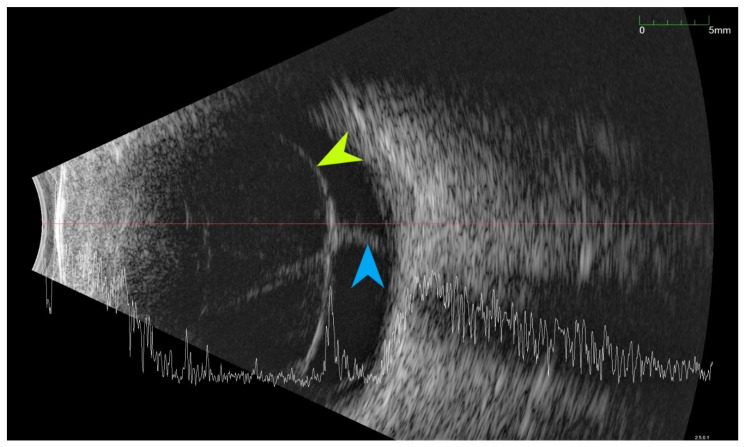
Ultrasound examination showed hyperechoic densities and point tractions on the retina (blue arrowhead). Densities forms strands and membranes with reduced mobility (green arrowhead). In order to confirm that the retina is not detached, the A-scan was superimposed over B-scan.

**Figure 10:**
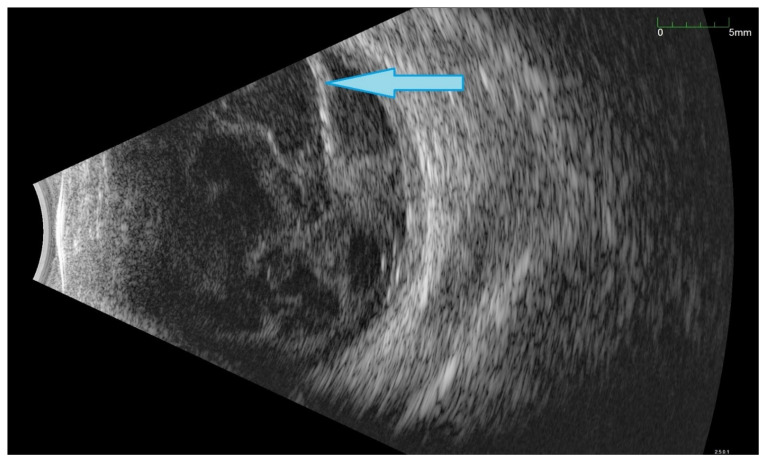
Ultrasound B scan with numerous hyperechoic densities in vitreous chamber. The blue arrow marks a detached retina. Hyperechoic masses are visible under the retina.

All of the above circumstances make the assessment of the vitreous body and retina in an ophthalmoscopic examination often impossible. The basic diagnostic tool in such cases is an ultrasound examination. Vitritis is inherent part of EE. Ultrasonography is nonspecific, however, it can indicate severity of the posterior involvement (Figure 6 and Figure 7) [80]. It also allows the assessment of the progression of changes (Figure 8). Features characteristic for EE include strands and membranes with reduced mobility (Figure 9). Other common changes are retinal detachment and subretinal abscess (Figure 10).

**Figure 11:**
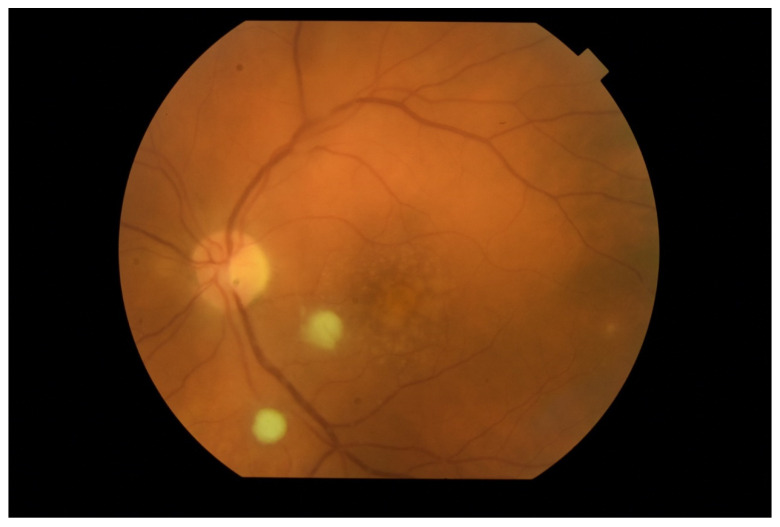
Fundus photography of the left eye. Two white “cotton-like” spots on the surface of the retina. Attention is also drawn to small foci along the blood vessels (“strings of pearls”).

**Figure 12:**
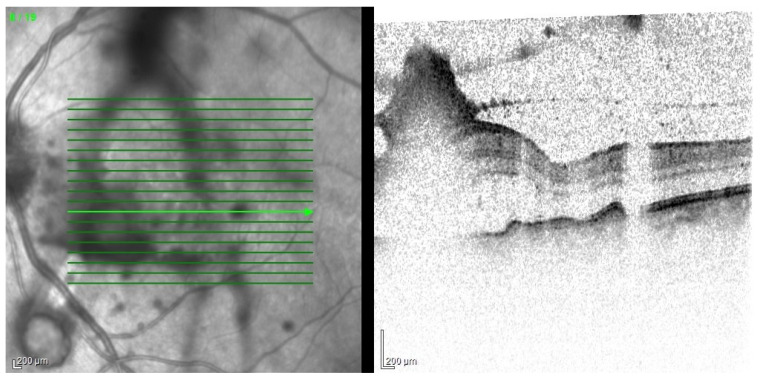
OCT examination of the patient presented in Figure 11. Scan through the retinal foci. There are also visible numerous densities in the vitreous.

Based on the clinical picture in the ophthalmological examination and the dynamics of the course of EE, certain conclusions about the etiology of the infection can be drawn. Bacterial infections proceed usually in acute while fungal infections in subacute manner. In fungal infections, characteristic cotton-like foci on the retina and in the vitreous body often appear, often in the form of “strings of pearls” (Figure 11). Optical coherence tomography enables fully accurate imaging of single inflammatory foci, as long as the transparency of the optical centers remains at an appropriate level (Figure 12).

The clinical diagnosis should be supported by a culture test. In the case of negative cultures, diagnostics can be aided by molecular techniques, in particular PCR. However, it is an expensive method and still not widely used. The test material may be aqueous fluid, vitreous, blood, and urine [1]. Importantly, only 75% of patients have a positive blood culture result [1]. However, case series in which the percentage of positive cultures is much lower are found in literature [4,81]. Additionally, the cultures of vitreous and aqueous humor can be unreliable, according to the available literature, the percentage of positive cultures varies from 60 to 90% depending on the sampling method [1,4]. In recent years, several case series have been published in which the percentage of positive cultures was very low. However, patients included in these statistics often received antibiotics before collecting the material for culture [18]. Blood or vitreous culture for patients already receiving treatment is warranted in the absence of a satisfactory response to therapy. In addition, when performing a vitrectomy, the vitreous is removed from the eye as a necessary part of the operation, so obtaining the material does not increase the risk to the patient in any way, as may be the case with a vitreous biopsy [82].

## 4. Treatment Possibilities

Treatment should be initiated as soon as possible. As the source of infection in EE is inside the body, systemic antibiotic therapy is recommended. Unfortunately, the distribution of drugs from the bloodstream to the eye is insufficient to control the intraocular infection [83]. The main route of administration of antibiotics in EE is intravitreal injection. In the past, periocular injections were also used, but nowadays such treatment is reserved for cases with abscesses of the sclera [84]. The most commonly used antibiotic for Gram-positive infections is vancomycin. Currently, ceftazidime is the most commonly used in gram-negative infections, less often amikacin and gentamicin [4].

In fungal infections, general treatment is usually sufficient in cases of chorioretinitis that proceed without lesions threatening the macular area. Macula-threatening chorioretinitis and cases of endophthalmitis require intravitreal antifungal injections in addition to systemic therapy. Usually, amphotericin or voriconazole is used. Vitrectomy is indicated in cases with significant vitritis. Fluconazole or voriconazole are preferred over amphotericin for the systemic treatment of sensitive isolates because azoles are less toxic and produce higher levels in the vitreous [1].

In more advanced cases, a vitrectomy should be considered, during which material for examination can be collected. The vitreous can also be collected for culture by biopsy, without the need for a vitrectomy. However, the vitrectomy procedure has additional benefits. Excision of the infected vitreous significantly reduces the population of microorganisms and the number of inflammatory cells in the eye. In more severe cases, eye can be fill with silicone oil, which not only inhibits the reproduction and spreads of pathogens, but also reduces inflammation [79,85].

Vitrectomy is not always possible in patients in a severe general condition. Treatment of life-threatening primary inflammation is always a priority. Moreover, for patients in severe general condition, the operation may potentially be too much of a burden. Then, the only available treatment is intravitreal injections of antibiotics. This method of treatment is widely used and effective. Serious side effects are rare. They include hemorrhage into the vitreous chamber, retinal tear, lens damage, temporary increase in intraocular pressure, and hemorrhagic occlusive retinal vasculitis [86,87]. In a meta-analysis of EE cases published between 2001 and 2012, 56% of patients received systemic antibiotics, 76% received intravitreal antibiotics, 12% received intravitreal corticosteroids, and 32% of eyes underwent vitrectomy. Performing a vitrectomy improved the prognosis of final visual acuity and decreased the risk of progression to the state requiring enucleation [88].

## 5. Complications

The prognosis for EE patients is generally poor. Final visual acuity depends on many factors, including promptness of appropriate therapy and the type of inflammatory pathogen. Very poor visual acuity (20/400 or worse) occurs in 40–90% of EE cases [1]. Up to 24% of patients require eyeball removal and mortality is up to 4% [4].

**Figure 13:**
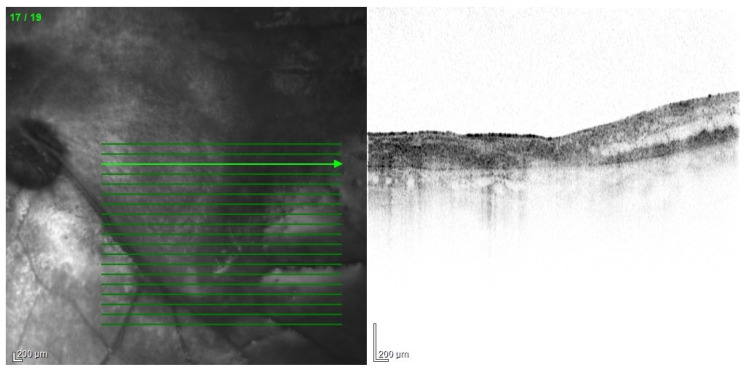
OCT scan with extensive structural changes in the retina. Visible atrophic changes, the remodeling of the layers of the retina, and intraretinal edema in the temporal part.

**Figure 14:**
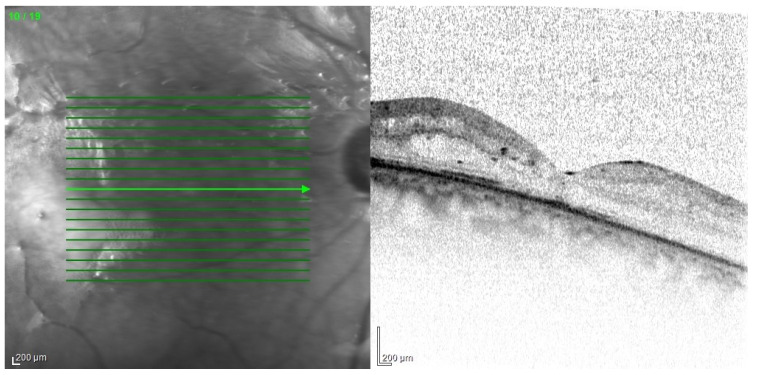
Condition after vitrectomy with silicone oil endotamponade. Persistent edema of the macular area seen on OCT.

The main problem in patients after endophthalmitis is the damage to the retina, especially atrophy and remodeling of retinal layers, which disrupts its function and worsening visual acuity [89]. Changes can be very accurately imaged in optical coherence tomography (Figure 13). In addition, dysfunction of the pigment epithelium and abnormalities of the blood–retinal barrier can lead to persistent macular edema (Figure 14).

**Figure 15:**
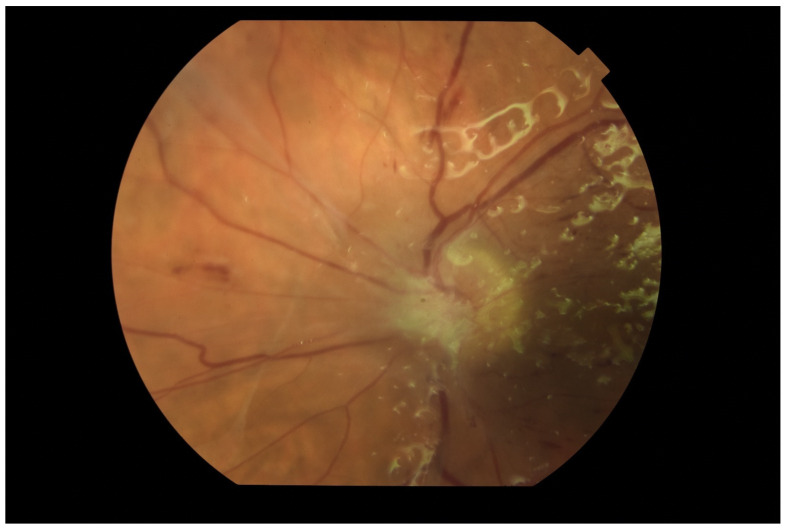
Photo of the optic nerve area, a vitreous chamber filled with silicone oil. Visible massive fibrous proliferation on the optic nerve disc and subretinal proliferations, lifting the retina.

**Figure 16:**
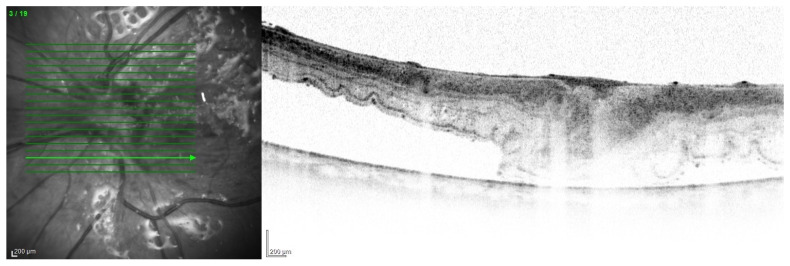
OCT examination of the patient presented in Figure 15. Cross-section of the optic disc area. Visible retinal elevation due to the presence of fibrosis.

**Figure 17:**
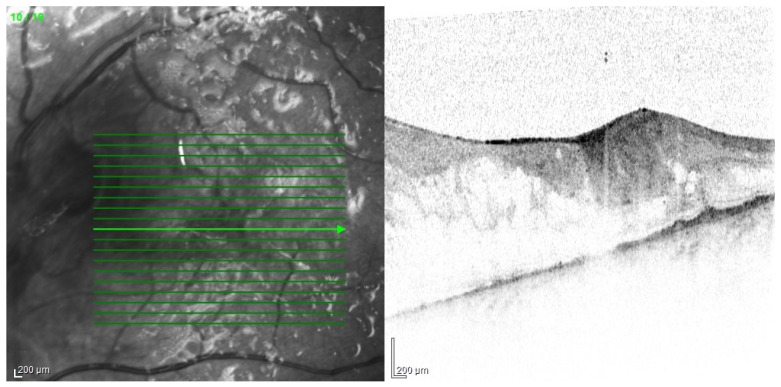
OCT examination of the patient presented in Figure 15. The macular area of the left eye. OCT shows tractional retinal folds, epiretinal membranes, cystic spaces, and disorganization of the retinal layers.

A much more serious complication of intense inflammation is the development of proliferative vitreoretinopathy. Membranes that make retina stiffer may lead to its tractional detachment. In advanced cases, it may be necessary to perform recurrent vitrectomy (Figure 15, Figure 16 and Figure 17).

**Figure 18:**
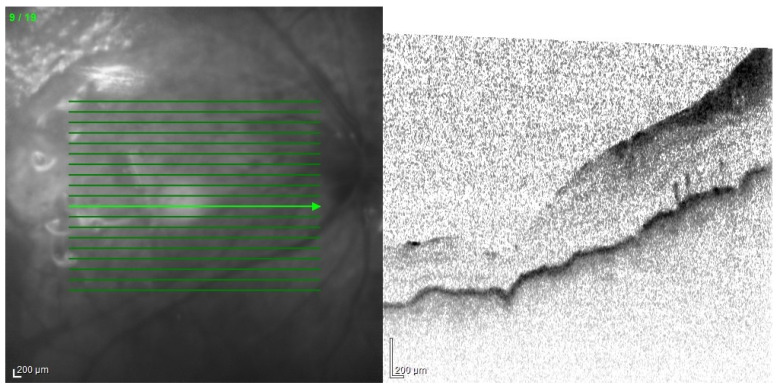
Typical picture of hypotony maculopathy in OCT-folds in the neurosensory retina and choroid.

In addition, vitrectomy patients usually require further surgeries to remove the silicone oil. Prolonged endotamponade can lead to oil emulsification and the resulting increase in intraocular pressure. A rarer but equally serious complication of EE is chronic hypotony. It causes hypotonic maculopathy which significantly reduces visual acuity. In the long term, it can even lead to eye atrophy (Figure 18).

## 6. Summary

Despite the enormous advances made in diagnostic and treatment modalities in recent years, EE remains a challenge. Diversified clinical picture, often rapid course of inflammation, difficulties in treatment, and poor prognosis are only some of the problems faced by ophthalmologists. Only a quick response and implementation of the correct treatment give the patient a chance to maintain a useful visual acuity. Even properly conducted therapy, if implemented too late, may not bring satisfactory results. Often the disease leads to loss of vision or enucleation. In the case of EE, an additional risk for the patient is the presence of a primary source of infection, which can be directly life-threatening.

## Data Availability

Not applicable.
